# Gut microbes on the risk of advanced adenomas

**DOI:** 10.1186/s12866-024-03416-z

**Published:** 2024-07-18

**Authors:** Zhuang Jing, Wu Zheng, Song Jianwen, Shen Hong, Yu Xiaojian, Wei Qiang, Yin Yunfeng, Wu Xinyue, Han Shuwen, Zhao Feimin

**Affiliations:** 1 Huzhou Central Hospital, Affiliated Central Hospital Huzhou University, Huzhou, Zhejiang Province China; 2 Fifth School of Clinical Medicine of Zhejiang Chinese Medical University (Huzhou Central Hospital), Huzhou, Zhejiang Province China; 3 Key Laboratory of Multiomics Research and Clinical Transformation of Digestive Cancer, Huzhou, Zhejiang Province China; 4 ICL, Junia, Université Catholique de Lille, Lille, France

**Keywords:** Advanced adenomas, Gut bacteria, Enteroviruses, Metagenomic sequencing, Prediction model

## Abstract

**Background:**

More than 90% of colorectal cancer (CRC) arises from advanced adenomas (AA) and gut microbes are closely associated with the initiation and progression of both AA and CRC.

**Objective:**

To analyze the characteristic microbes in AA.

**Methods:**

Fecal samples were collected from 92 AA and 184 negative control (NC). Illumina HiSeq X sequencing platform was used for high-throughput sequencing of microbial populations. The sequencing results were annotated and compared with NCBI RefSeq database to find the microbial characteristics of AA. R-vegan package was used to analyze α diversity and β diversity. α diversity included box diagram, and β diversity included Principal Component Analysis (PCA), principal co-ordinates analysis (PCoA), and non-metric multidimensional scaling (NMDS). The AA risk prediction models were constructed based on six kinds of machine learning algorithms. In addition, unsupervised clustering methods were used to classify bacteria and viruses. Finally, the characteristics of bacteria and viruses in different subtypes were analyzed.

**Results:**

The abundance of *Prevotella sp900557255*,* Alistipes putredinis*, and *Megamonas funiformis* were higher in AA, while the abundance of *Lilyvirus*, *Felixounavirus*, and *Drulisvirus* were also higher in AA. The Catboost based model for predicting the risk of AA has the highest accuracy (bacteria test set: 87.27%; virus test set: 83.33%). In addition, 4 subtypes (B1V1, B1V2, B2V1, and B2V2) were distinguished based on the abundance of gut bacteria and enteroviruses (EVs). *Escherichia coli D*, *Prevotella sp900557255*,* CAG-180 sp000432435*,* Phocaeicola plebeiuA*,* Teseptimavirus*, *Svunavirus*, *Felixounavirus*, and *Jiaodavirus* are the characteristic bacteria and viruses of 4 subtypes. The results of Catboost model indicated that the accuracy of prediction improved after incorporating subtypes. The accuracy of discovery sets was 100%, 96.34%, 100%, and 98.46% in 4 subtypes, respectively.

**Conclusion:**

*Prevotella sp900557255* and *Felixounavirus* have high value in early warning of AA. As promising non-invasive biomarkers, gut microbes can become potential diagnostic targets for AA, and the accuracy of predicting AA can be improved by typing.

**Supplementary Information:**

The online version contains supplementary material available at 10.1186/s12866-024-03416-z.

## Highlights

The bacteria (including *Prevotella sp900557255*, *Alistipes putredinis*, *Megamonas funiformis*, etc.*)* and viruses (including *Lilyvirus*, *Felixounavirus*, and *Drulisvirus*, etc.) existed differences in AA. And there were correlations between AA and basic information, lipid index and serological index.

Prediction models based on bacteria and viruses were established to distinguish AA, and the accuracy reached 87.27% and 83.33%.

A new typing method was established based on bacteria and viruses to divide gut microbes into 4 subtypes.

Prediction models after typing had higher accuracy (100% in B1V1, 96.34% in B1V2, 100% in B2V1, 98.46% in B2V2).

**Supplementary Information:**

The online version contains supplementary material available at 10.1186/s12866-024-03416-z.

## Introduction

Colorectal adenomatous polyps or adenomas are common precancerous lesions and are the origin of most colorectal adenocarcinoma cases [1]. Adenomas are common in the adult population, occurring in about 20-40% of people over the age of 50 [2]. Advanced adenoma (AA) is one kind of adenomas with a diameter of 10 mm or greater. Adenomas less than 10 mm in diameter that contain more than 25% villi or are highly atypical hyperplasia or adenocarcinoma can also be classified as AA. There is considerable evidence that patients with AA are at increased risk of advanced tumors, including CRC [3, 4]. AAs are the earliest detectable initiating event of CRC, and the vast majority of CRC originates from AA [5, 6]. At present, there are many detection methods for CRC, but the detection methods for AA are relatively limited. Given that early symptoms of AA are often inconspicuous, the sole available early screening method is colonoscopy [7]. However, colonoscopy is an invasive procedure with low patient compliance. Consequently, the search of new prediction targets is of paramount importance.

The gut microbe is an independent and complex microecology system. The interaction between different gut bacteria can maintain the homeostasis of gut microenvironment and participate in host metabolism, substance absorption, and transformation [8, 9]. The changes in specific bacterial abundance in the gut microenvironment have been used as biomarkers for screening gastrointestinal diseases, including inflammatory bowel disease (IBD), CRC, and irritable bowel syndrome (IBS). For example, *Bifidobacterium*, *Porphyromonas*, *Clostridium*, *Trichospiridae*, *Prevotella*, *Fecal bacteria*, *Ruminococcus*, and *Lactobacillus* constitute the most prominent and influential taxonomic group together in IBD [10–12]. Research has determined that an overabundance of *F. nucleatum* is associated with poor prognosis in metastatic CRC [13]; Colibactin-producing *E. coli*,* Enterococcus faecalis*, Enterotoxin-producing *Bacteroides fragilis*, *Streptococcus bovis*, and *Peptostreptococcus anaerobius* are positively associated with CRC [14, 15]. In addition, enteroviruses (EVs) are a large genus of positive single stranded RNA viruses, and their interactions can cause many important and widespread human diseases [16]. Viral homeostasis disorders may cause or promote inflammatory diseases (such as IBD), promote dysplasia, and eventually lead to cancer with severe symptoms and high mortality [17]. Studies have shown that viruses can infect human cells and mutate and are therefore directly involved in inflammation and tumorigenesis [18, 19]. And the results of Fatemeh et al. indicated that the EVs exhibits the potential for autonomous immune regulation [20]. Studies have shown that patients of CRC contained significantly higher proportions of *Bacteroidaceae*, *Oscillospiraceae*, and *Peptostreptococcaceae phages*. Considered together, both gut bacteria and EVs play important roles in the occurrence and development of CRC. Since the vast majority of CRC originates from AA, the study of gut microbes’ differences in AA may provide a new and more accurate basis for the treatment and risk assessment of AA and the prevention of CRC.

Fecal metagenomics is a useful tool for the quantification of gut microbiomes [21]. Metagenomic second-generation sequencing (mNGS) represents a comprehensive method by which almost all potential pathogens, including viruses, bacteria, fungi, and parasites, can be accurately identified in a single measurement [22]. By unbiased sequencing of all nucleic acids in the sample using mNGS, readings can be calculated and classified to identify different microorganisms [23].

In this study, metagenomic sequencing was used to conduct gene sequencing on fecal samples of AA and NC and analyzed differential genes of the gut microbiome between them. The differences in the abundance and community structure of gut microbes were described and compared. Furthermore, the correlation between gut microbes and diseases in AA was analyzed. This study constructed the best risk prediction models based on different bacteria and different viruses to identify AA. Moreover, gut microbes were typed by unsupervised clustering, and then prediction models of 4 subtypes were constructed, and the characteristics of bacteria and viruses in different subtypes were analyzed. The subtypes could provide an alternative method for the treatment and prevention of AA.

## Methods

### Samples

#### Inclusion and exclusion criteria

A total of 92 patients with AA and 184 healthy individuals were recruited in our study. Healthy volunteers who had no lesions by colonoscopy were classified as the negative control (NC). The inclusion criteria for AA were as follows: ① Patients with progressive adenoma diagnosed by pathological examination and volunteered to participate in the study. ② Patients between 45 and 75 years old.

The exclusion criteria were as follows: ① Patients with CRC and other gut diseases, such as ulcerative colitis and Crohn’s disease. ② Patients with malignant tumors in other sites. ③ Patients with diarrhea and constipation. ④ Patients with fever caused by bacterial or viruses. ⑤ Patients with a medicine history of oral microbial agents or antibiotics within the last 2 months. ⑥ Patients with known primary organ failure.

Patients at Huzhou Central Hospital from January 2019 to December 2022 were studied. The clinical protocols involving the patients and the informed consent form were approved by the Ethics Committee of Huzhou Central Hospital (no. 20191101-01) and the Chinese Clinical Trial Registry (http://www.chictr.org.cn; ChiCTR2000034061). All subjects signed informed consent under the guidelines approved by the Ethics Committee of Huzhou Central Hospital.

#### Collection of fecal samples and clinical information

Basic patient information, clinical indicators, and pathological data were obtained from the medical record management system of Huzhou Central Hospital and informed consent was also signed by patients. Fecal samples were collected in the morning prior to breakfast. Approximate 5 to 10 g fecal samples were obtained after defecation without the use of a gut microbiome purgative or lubricant. Within half an hour, the fecal samples were stored in an ultralow-temperature freezer. The sample preservation time was not beyond 1 month. The basic characteristics of these patients were shown in Table 1. There were no differences in diet and lifestyle among all samples.

**Table 1:** Characteristics of study participants

		NC (*n* = 184)	AA (*n* = 92)	*P*
Gender	Male	107	44	0.104
	Female	77	48	
Age		67.92 ± 7.26	66.58 ± 8.73	0.701
BMI		25.05 ± 10.79	23.18 ± 10.88	0.506
WBC		5.77 ± 4.91	5,41 ± 2.40	0.326
Albumin		39.10 ± 19.05	41.97 ± 13.79	0.266
TG		1.33 ± 0.80	1.46 ± 1.92	0.528
TC		4.43 ± 2.05	4.75 ± 5.97	0.322
HDL		48.68 ± 23.41	52.79 ± 25.70	0.386
LDL		88.23 ± 45.47	101.36 ± 43.65	0.450

Totally, 92 fecal samples of AA and 184 fecal samples of NC were selected based on the quantity of data obtained. Clinical information, including age, sex, BMI, lipids, and serological indicators, was collected from the selected samples.

### Acquisition of microbial genomes

According to the manufacturer’s agreement, E.Z.N.A^®^ fecal DNA sampler (Omega Bio tek, Norcross, GA, USA) was used to extract microbial DNA from fecal samples. The specific methods refer to our previously published article [24]. All amplicon sequencing was performed by Shanghai Biozeron Lingen Biotechnology Co. Ltd (Shanghai, China).

Through the quality control of the reads, the human genome (version: hg19) was then mapped using the BWA mem algorithm (parameters: - M − 32 k - t 16, http://bio-bwa.sourceforge.net/bwa.shtml). Reads that remove host genome contamination and low-quality data were called clean reads for further analysis. The clean read classification for each sample was determined by Kraken2 [25] using a customized Kraken database. DNA viruses and DNA bacteria databases were selected as annotation database. All reads were classified into seven phylogenetic levels (domain, phylum, class, order, family, genus, and species) or unclassified. Bracken (https://ccb.jhu.edu/software/bracken/) that estimated the species abundance could accurately calculate the level of abundance, even in the closer to the same species.

### Bioinformatics analysis

The α diversity analysis was conducted to reveal the diversity indices, including the richness and Shannon diversity indices. The β diversity analysis was performed using the community ecology package, R-vegan package. Gephi was applied to visualize the relationship between gut microbes and clinical information through correlation heatmap. PCA, PCoA and NMDS analyses were conducted using the Vegan R package. For the identification of biomarkers for highly dimensional bacteria, linear discriminant analysis effect size (LDA Effect Size, LEfSe) analysis was done [26]. Student’s t test was performed to examine the changes and dissimilarities among classes followed by LDA analysis to determine the size effect of each distinctively abundant taxa [27]. Unsupervised clustering was carried out by R software (Version:4.3.1). ConsensusClusterPlus package was employed to perform consensus clustering on gut bacterial metagenomics data. This clustering divided the samples into subtypes, which facilitated subsequent comparative analyses among different subtypes. The specific parameters used included maxK = 9, reps = 50, pItem = 0.8, pFeature = 1, distance = “euclidean”, and clusterAlg = “km”. The probably approximately correct (PAC) algorithm was used to select the best K value to optimize the clustering model.

### Multiple machine learning models in the prediction of AA

AA prediction models were built based on the differential bacteria and viruses before and after gut typing. After the typing of the gut via unsupervised clustering, the total sample was divided into 4 sections and differential bacteria and viruses were inconsistent in 4 subtypes. According to the classification conditions, we chose the differential bacteria and viruses corresponding to different samples size to build prediction models, among which 70% were used as the discovery set and 30% as the test set. Subsequently, the logistic regression (LR) model, the random forest (RF) model, the neural network (NN) model, the CatBoost model, the gradient boosted decision tree (GBDT) model, and the support vector machine (SVM) model were used to construct the model. The methods of model construction were the same as previously described [28, 29].

## Results

### Characteristics of gut bacteria in AA

**Fig. 1:**
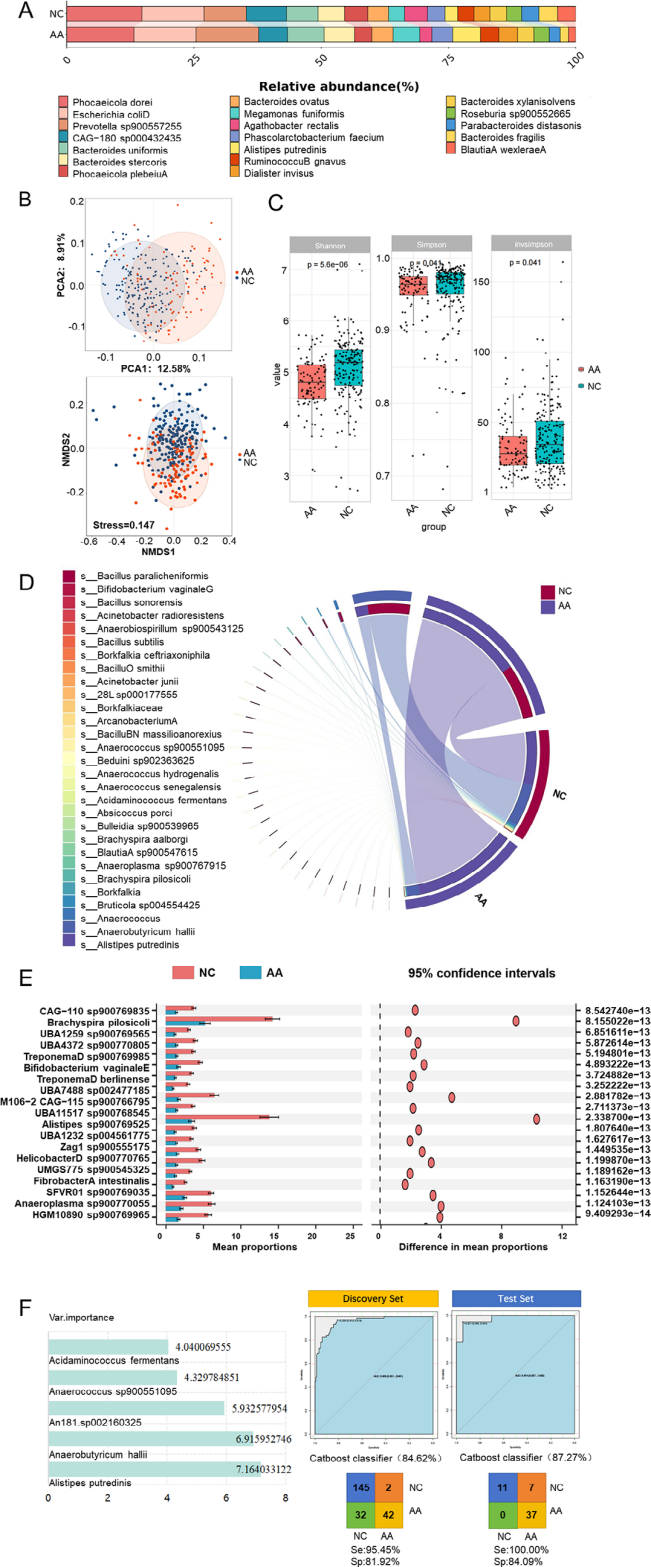
Gut bacteria in AA: Descriptive analysis, diversity analysis, differential analysis, correlation analysis and modeling were used for gut bacteria between AA and NC: Histogram of the bacterial relative abundance (**A**), NMDS and PCA analysis (**B**), Box diagram (**C**), Chord chart (**D**), T-test (**E**), and CatBoost model (**F**)

Species abundance difference analysis was performed to compare the differences in gut bacteria at the species level in AA. At the species level, the top 5 abundant gut bacteria in AA were *Phocaeicola dorei*, *Prevotella sp900557255*, *Escherichia coliD*, *Bacteroides uniformis*, and *Bacteroides stercoris.* The top 5 abundant gut bacteria in NC were *Phocaeicola dorei*,* Escherichia coliD*,* Prevotella sp900557255*,* CAG-180 sp000432435*, and *Bacteroides uniformis* (Fig. 1A). The samples were clustered according to gut bacteria, and it was found that the two groups had their own clustering ranges (Fig. 1B). As depicted in Fig. 1C, the Simpson index and Invsimpson index of gut bacteria in AA and NC exhibited similarity, while the Shannon index revealed significant disparity in the alpha diversity of gut bacteria among AA and NC. Considering the impacts of the interaction between the gut microbe and the host on AA, the correlation between the host’s disease status and the gut microbe was analyzed (Fig. 1D). *Alistipes putredinis* has the highest correlation with AA and NC, and it has a greater correlation with AA. *Anaerobutyricum hallii* and *Anaerococus* also have a certain correlation with AA and NC, and the correlation is greater with NC. Further t-tests were carried out to compare and screened 20 differential gut bacteria, including *CAG-831 sp900769885*, *UBA1259 sp900770685*, and *UMGS1820 sp900769795* (Fig. 1E). Six risk prediction models were constructed based on screened different bacteria, including LR model, RF model, NN model, CatBoost model, GBDT model (Figure S2). The CatBoost model (Fig. 1F) had the best modeling effect among these models (AUC: 0.956, specificity: 95.45% sensitivity: 81.92%, accuracy: 84.62%), and it also had the best modeling performance in the test set (AUC: 0.974, specificity:100%, sensitivity: 84.09%, accuracy: 87.27%).

### Characteristics of enteroviruses in AA

**Fig. 2:**
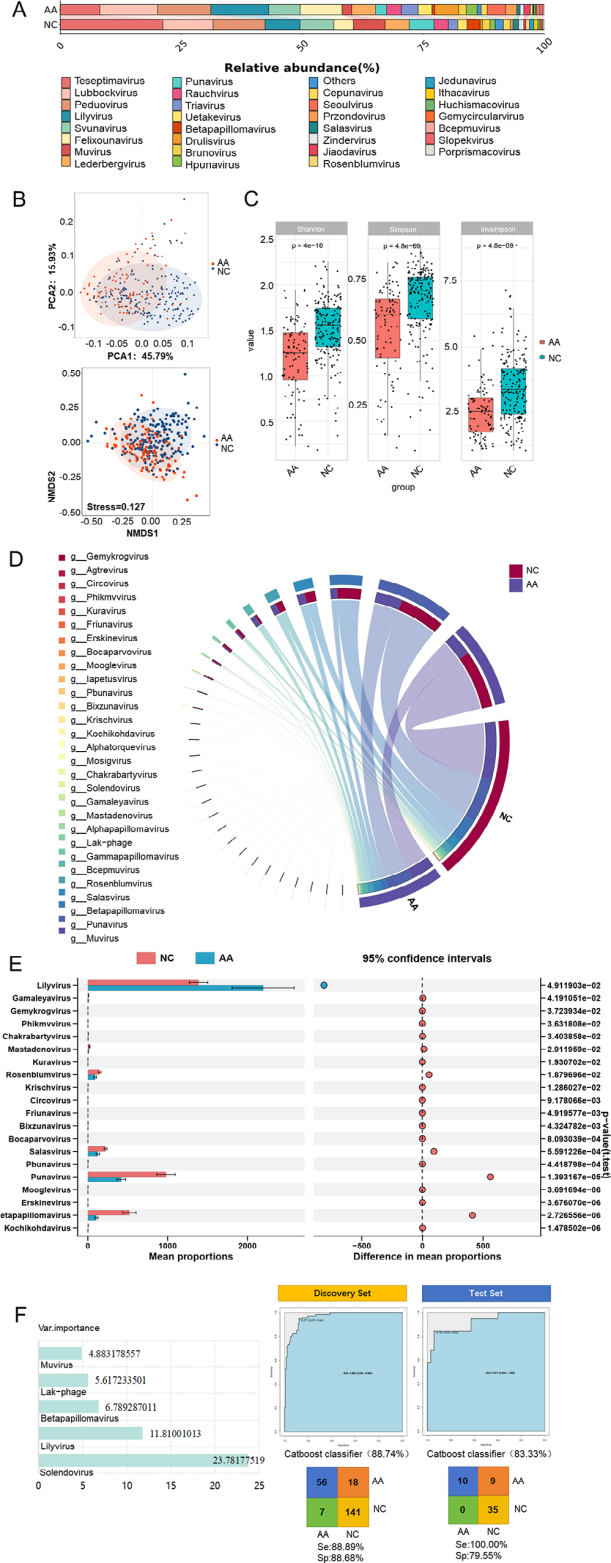
EVs in AA: Descriptive analysis, diversity analysis, differential analysis, correlation analysis and modeling were used for EVs between AA and NC: Histogram of the viral relative abundance (**A**), NMDS and PCA analysis (**B**), Box diagram (**C**), Chord chart (**D**), T-test (**E**), and CatBoost model (**F**)

The result showed that the top 5 EVs in AA and NC were consistent, namely *Lilyvirus*, *Lubbockvirus*, *Peduovirus*, *Felixounavirus*, *Teseptimavirus* (Fig. 2A). Similarly, the samples were clustered according to EVs, and it was also found that the two groups had their own clustering ranges (Fig. 2B). As depicted in Fig. 2C, the Shannon index, Simpson index and Invsimpson index all revealed significant disparity in the alpha diversity of EVs among AA and NC. According to the chord diagram, *Muvirus* was more correlated with NC and AA, and its correlation with NC was greater (Fig. 2D). Moreover, *Punavirus*, *Betapapilomavirus*, and *Salasvirus* also had a certain correlation with AA and NC, and the correlation with the NC is greater. Through t-test comparison and analysis, 20 differential EVs, including *Muvirus*, *Mosigvirus*, and *Iapetusvirus*, were screened (Fig. 2E). Besides, 6 risk prediction models were established based on characteristic EVs, and the CatBoost model had a good modeling effect as well (Figure S3). The results proved that the AUC of the model based on EVs was 0.962; the specificity and sensitivity were 88.89% and 88.68%, respectively; the accuracy was 88.74% (Fig. 2F). The AUC, accuracy, sensitivity, and specificity of the test set is 0.917, 83.33%, 100%, and 79.55%, respectively.

### Correlation between gut microbe and clinical information

**Fig. 3:**
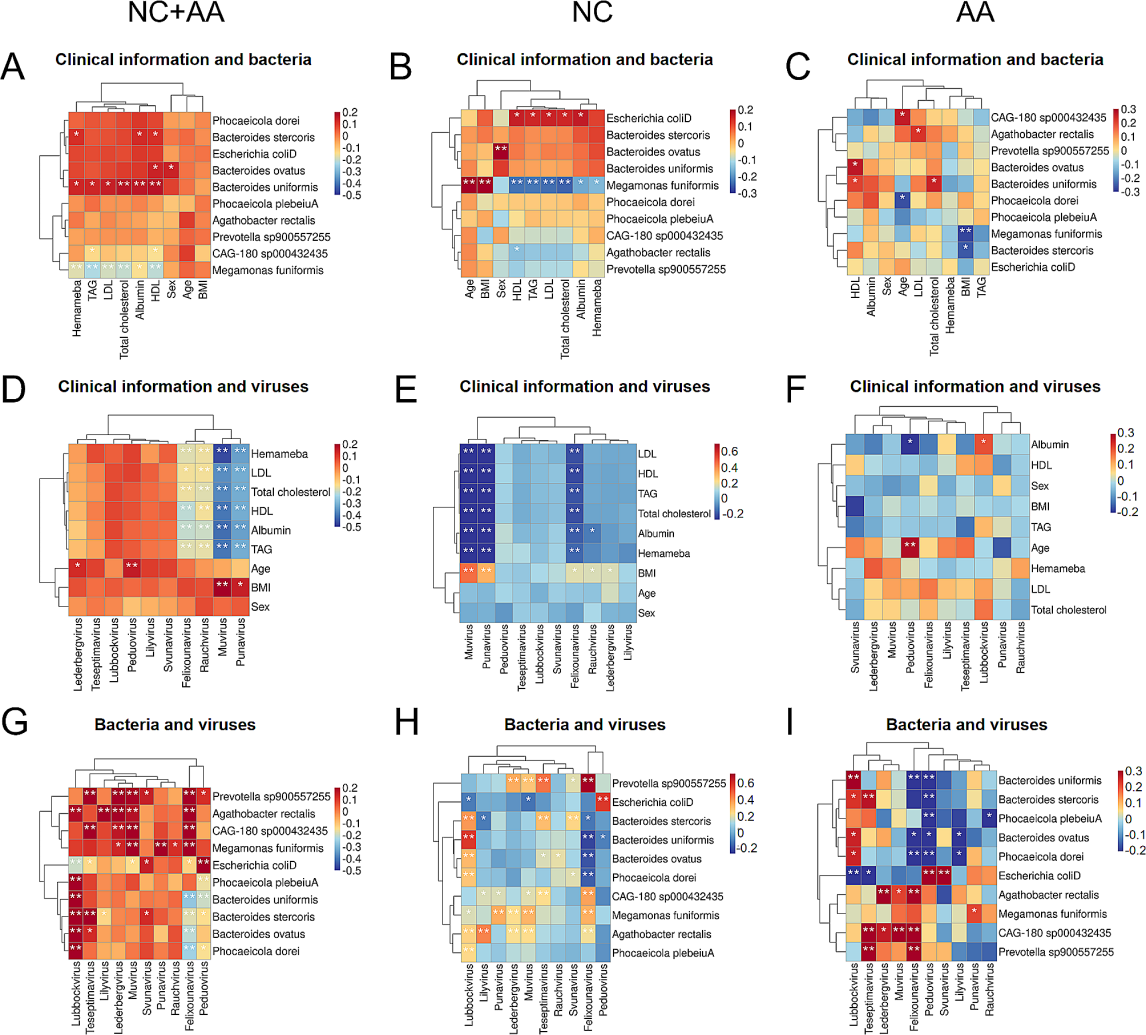
Correlation among gut bacteria, EVs, and clinical information: Correlation heat maps were used to show the relationship between gut microbes and clinical information in different groups, including NC + AA group, NC group and AA group: Heatmaps of gut bacteria (**A-C**), Heatmaps of EVs (**D-F**), and Heatmaps of gut bacteria and viruses (**G-I**).

In order to analyze the interaction between gut bacteria, EVs, and clinical information in AA and NC, different gut bacteria and EVs were included for intra-group correlation analysis. The results of total sample analysis showed that *Bacteroides uniformis* and *Megamonas funiformis* were related to clinical information in the group of bacteria (Fig. 3A), *Mivirus*,* Punavirus*,* Rauchvirus*, and *Felixounavirus* were significantly related to clinical information in the group of viruses (Fig. 3D). For example, there was a significant negative correlation between *Mivirus* and Hemameba (*r*=-0.46, *p* < 0.05), and a significant positive correlation between *Mivirus* and BMI (*r* = 0.23, *p* < 0.05). According to the results among gut bacteria, *Megamonas funiformis* had a high correlation with clinical information in NC (Fig. 3B), while it only had a high correlation with BMI in AA (Fig. 3C, *r*=-0.28, *p* < 0.05). Moreover, there was a negative correlation between *Megamonas funiformis* and BMI in AA (Fig. 3C) while it was negatively correlated with BMI in NC (Fig. 3B, *r* = 0.23, *p* < 0.05). Among EVs, *Muvirus*, *Peduovirus*, and *Felixounavirus* had a higher correlation with clinical information in NC (Fig. 3E), while their correlation was weaker in AA (Fig. 3F). Moreover, *Peduovirus* was poorly correlated with age in NC (Fig. 3E, *r* = 0.07, *p* < 0.05), while it was positively correlated with age in AA (Fig. 3F, *r* = 0.28, *p* < 0.05).

Further analysis of the relationship between bacteria and viruses showed that *Lubbockvirus* and *Felixounavirus* were closely related to a variety of bacteria (Fig. 3G). Among them, the positive correlation between *Lubbockvirus* and *Bacteroides uniformis* (*r* = 0.57, *p* < 0.05) and the positive correlation between *Felixounavirus* and *Prevotella sp900557255* (*r* = 0.77, *p* < 0.05) were the most prominent. For the relationship between EVs and gut bacteria, *Escherichia coli D* was negatively correlated with all EVs except *Peduovirus* (*r* = 0.56, *p* < 0.05) in NC (Fig. 3H), while in AA, there was also a significant positive correlation with *Svunavirus* (*r* = 0.30, *p* < 0.05) and *Peduovirus* (Fig. 3I, *r* = 0.53, *p* < 0.05). Among NC, the correlation between *Lubbockvirus* and bacteria was higher, while the correlation between *Peduovirus* and bacteria was weaker (Fig. 3H). On the contrary, in AA, *Peduovirus* had a higher correlation with bacteria, while *Lubbockvirus* had a weaker correlation with bacteria (Fig. 3I).

### Typing and analysis of gut microbe

**Fig. 4:**
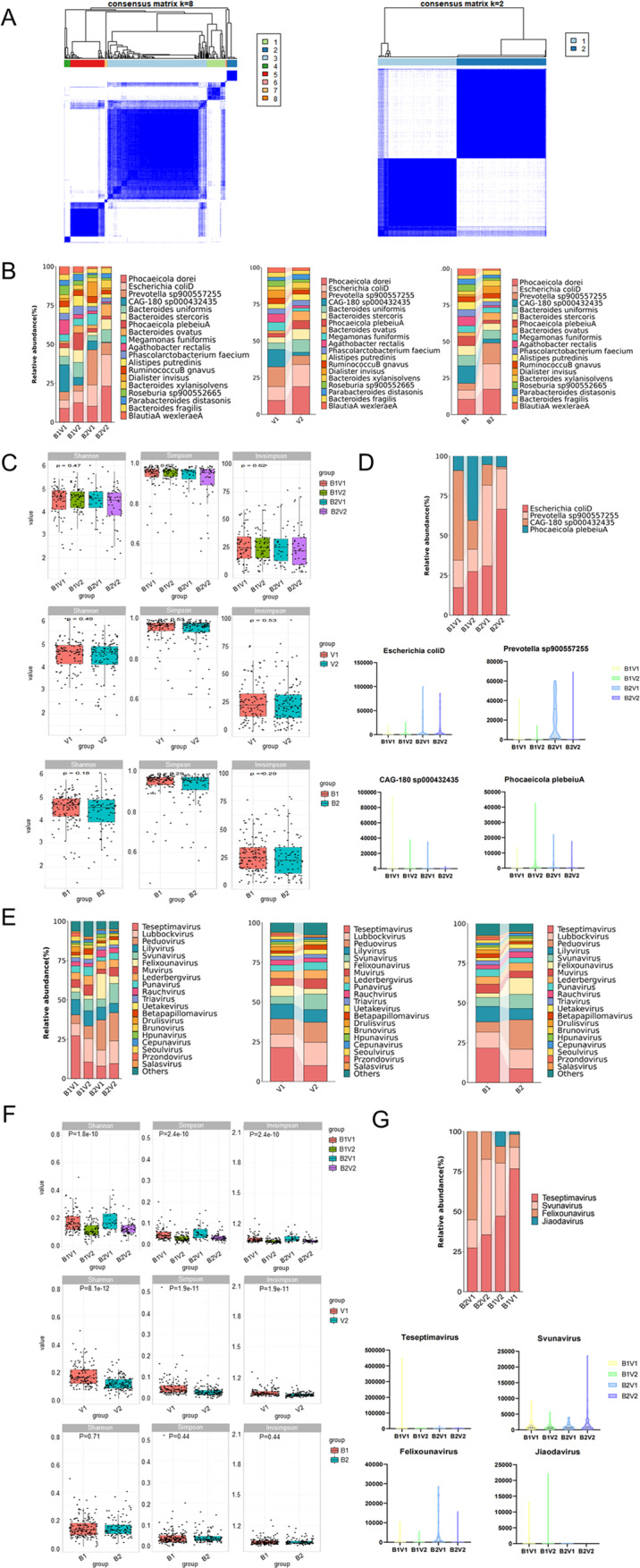
Typing and analysis of gut microbes: Differential gut bacteria and viruses were used for gut typing, and finally 4 subtypes were obtained via unsupervised clustering. The proportions of gut bacteria and viruses were analyzed, and gut microbes with the most significant differences in proportions were screened among 4 subtypes: Unsupervised clustering (**A**), Stacked bar graph of the top 20 abundant gut bacteria in the 4 subtypes, 2 viral subtypes and 2 bacterial subtypes (**B**), Box diagram of the top 20 abundant gut bacteria in the 4 subtypes, 2 viral subtypes and 2 bacterial subtypes (**C**), 4 bacteria with most significant differences in proportion among 4 subtypes (**D**), Stacked bar graph of the top 20 abundant gut viruses in the 4 subtypes, 2 viral subtypes and 2 bacterial subtypes (**E**), Box diagram of the top 20 abundant EVs in the 4 subtypes, 2 viral subtypes and 2 bacterial subtypes (**F**), and 4 viruses with most significant differences in proportion among 4 subtypes (**G**)

Gut bacteria and EVs were classified by unsupervised clustering (Fig. 4A), and the results revealed that gut bacteria were clustered into 8 categories and EVs were clustered into 2 categories (Tables 2 and 3). According to the proportion of AA and NC, the ratio greater than 0.5 was divided into susceptible types (B1, V1), and the ratio less than 0.5 was divided into nonsusceptible types (B2, V2). Finally, it was classified into 4 subtypes, namely B1V1, B1V2, B2V1, and B2V2. The proportion of AA in the B1V1 subtype was 0.45, in B1V2 subtype was 0.28, in B2V1 subtype was 1.63, and the proportion in B2V2 subtype was 0.48 (Table 4). Afterwards, relative abundance maps were performed on the top 20 gut bacteria and the top 30 EVs in 4 subtypes. The abundance of *Escherichia coliD*, *Prevotella sp900557255*, *CAG − 180 sp000432435*, and *Phocaeicola plebeiuA* were higher in 4 subtypes distinctly (Fig. 4B). However, in pairwise comparisons between groups, there were no significant differences (Fig. 4C). *Escherichia coliD* was higher in B1V1 subtype, *Prevotella sp900557255* was higher in B1V2 subtype, *CAG − 180 sp000432435* was higher in B2V1 subtype, and *Phocaeicola plebeiuA* was higher in B2V2 subtype (Fig. 4D). Similarly, the abundance of *Teseptimavirus*, *Lubbockvirus*, *Peduovirus*, and *Lilyvirus* were higher in 4 subtypes distinctly (Fig. 4E). As depicted in Fig. 4F, the Simpson index, Shannon index, and Invsimpson index revealed significant disparities in the alpha diversity of EVs across the 4 subtypes and virus subtypes. However, bacteria subtypes did not show significant differences. *Teseptimavirus* was higher in B1V1 subtype, *Felixounavirus* was higher in B2V1 subtype, and *Svunavirus* was higher in B2V2 subtype (Fig. 4G). The differences in the abundance of the 4 bacteria and 4 viruses may be the basis for unsupervised clustering.

**Table 2:** Unsupervised clustering results of gut bacteria

Category	NC	AA	SUM	AA/NC
1	13	16	29	1.23
2	9	7	16	0.78
3	116	43	159	0.37
4	8	2	10	0.25
5	34	21	55	0.62
6	1	1	2	1.00
7	2	2	4	1.00
8	1	0	1	0.00
SUM	184	92	276	

**Table 3:** Unsupervised clustering results of EVs.

Category	NC	AA	SUM	AA/NC
1	76	53	129	0.70
2	108	39	147	0.36
SUM	184	92	276	

**Table 4:** Distribution of NC and AA in four subtypes

Subtype	NC	AA	SUM	AA/NC
B1V1	60	27	87	0.45
B1V2	64	18	82	0.28
B2V1	16	26	42	1.63
B2V2	44	21	65	0.48
SUM	184	92	276	

### Differential analysis and modeling of four subtypes of gut microbe

**Fig. 5:**
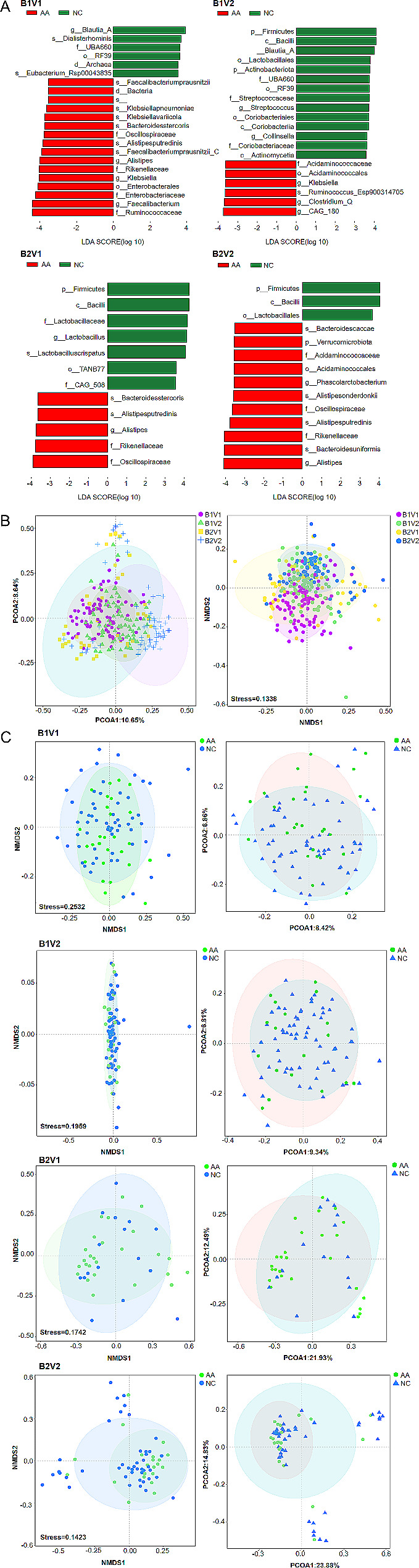
Differential analysis of four subtypes: Based on gut typing, the differential bacteria and viruses between AA and NC were screened: LDA analysis of 4 subtypes (**A**). Differences in diversity among the 4 subtypes and the diversity of the 4 subtypes between AA and NC were analyzed: NMDS and PCOA analysis in the 4 subtypes (**B**), NMDS and PCOA analysis of 4 subtypes between AA and NC (**C**)

Totally, 22 different bacteria in the B1V1 subtype, 20 different bacteria in the B1V2 subtype, 12 different bacteria in the B2V1 subtype, and 14 different bacteria in the B2V2 subtype were selected. The results indicated that *Firmicutes*, *Bacilli*, and *Lactobacillales* were significantly reduced in AA, while *Oscillospiraceae*, *Rikenellaceae*, and *Alistipes* were significantly increased in AA. This provided potential biological markers for risk prediction of AA (Fig. 5A). The samples were clustered according to the gut microbes, and the 4 subtypes were found to have their own clustering ranges (Fig. 5B). The β-diversity of gut bacteria of NC and AA in B1V1, B1V2, B2V1, and B2V2 were also analyzed by NMDS and PCOA (Fig. 5C). PCOA analysis found that among the 4 subtypes, B2V2 showed the most significant difference in clustering effect between AA and NC.

**Fig. 6:**
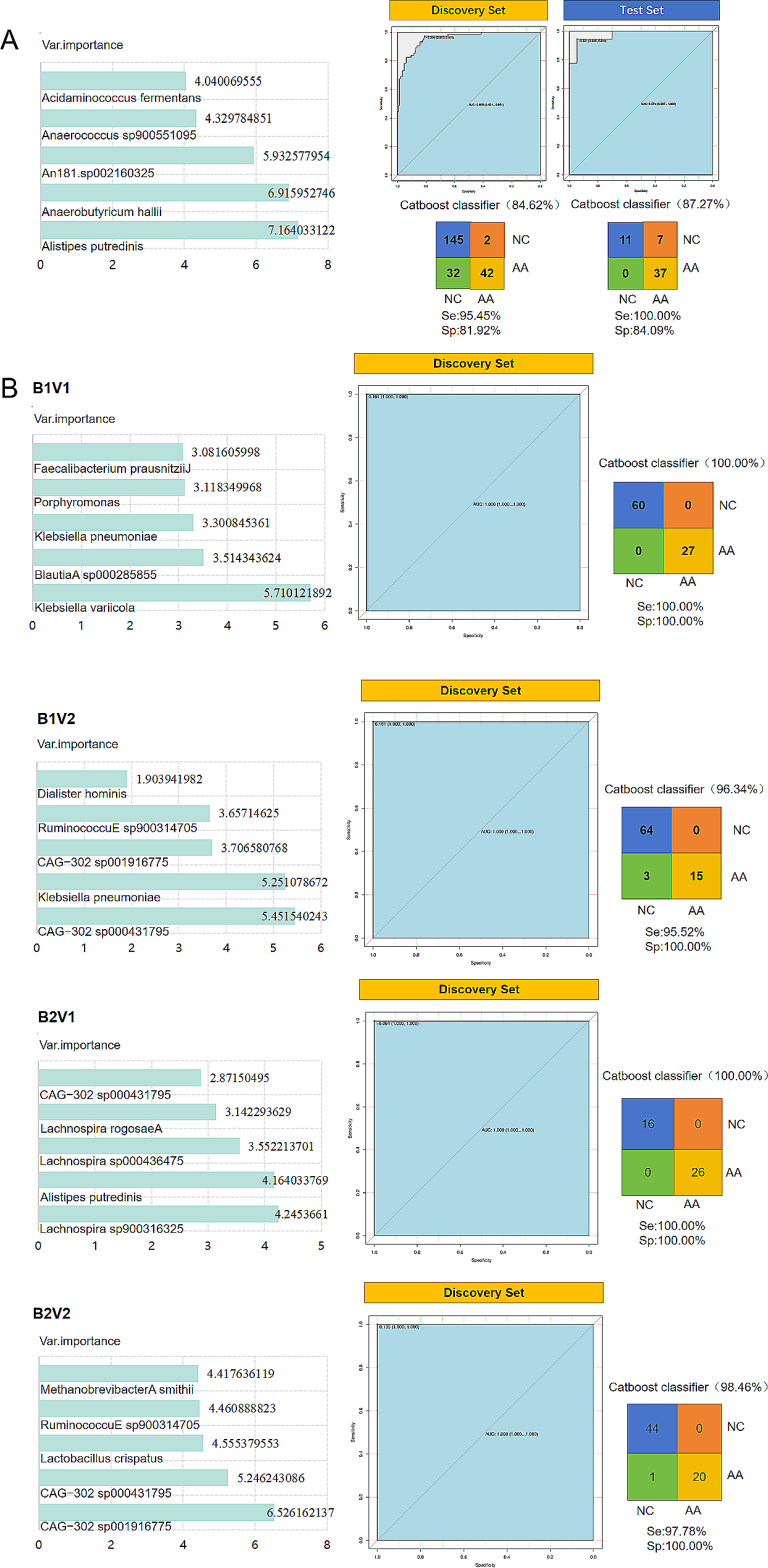
Modeling based on specific gut bacteria after typing: CatBoost models based on differential gut bacteria were built: Before typing (**A**), After typing (**B**)

CatBoost models were used to establish risk prediction models based on characteristic bacteria in B1V1, B1V2, B2V1, and B2V2 4 subtypes (Fig. 6B). The results were shown in Table 5, which indicated that the model after typing had higher accuracy and better performance than those before classification so that it had a better prediction effect.

**Table 5:** Discovery set results of modeling based on specific gut bacteria after typing

Subtype	AUC	Accuracy	Sensitivity	Specificity
B1V1	1	100%	100%	100%
B1V2	1	96.34%	95.52%	100%
B2V1	1	100%	100%	100%
B2V2	1	98.46%	97.78%	100%

## Discussion

CRC follows a stereotyped progression from normal to atypical to carcinoma, and AAs are the earliest detectable initiating event of CRC [30]. The causal relationship between gut microbes and various diseases has become a hot topic in recent years, and various diseases are related to gut microbe imbalance. The colonoscopy results of the patients showed NCs were more than AAs, so we recruited volunteers according to 2:1. A total of 184 NCs and 92 AAs were recruited in our study. Metagenomics sequencing technology was used to sequence fecal samples. The analysis revealed that gut bacteria and EVs existed differences in AA. Prediction models with gut microbes were established to distinguish AA, and the accuracy reached 83.33%. Besides, a new typing method was established based on bacteria and viruses to divide gut microbes into 4 subtypes. In the end, we found that prediction models after typing had higher accuracy.

One of the earliest studies to detect the microbiota associated with AA compared the gut microbiota of patients with and without adenomas. Rectal mucosal biopsy showed the enrichment of *Proteobacteria* in adenoma samples through 16 S rRNA sequencing, with a relative increase in *Faecalibacterium* and *Dorea bacteria*, and a relative decrease in *Bacteroides* and *Coprococcus* [31]. In our study, we found that NC had higher richness and diversity of gut bacteria compared with AA, and *Prevotella sp900557255*, *Alistipes putredinis*, and *Megamonas funiformis* were relatively abundant in AA. Recent studies made by Zhong et al. demonstrated that, compared with healthy individuals, patients with adenomas had higher abundance of *Bacteroides*,* Prevotella-2*, and *Agathobacter* in their feces, while the abundance of *Haemophilus*,* Escherichia Shigella*,* Fusobacterium*, and *Streptococcus* is lower [32], which is broadly in line with our findings. As a key factor in balancing health and disease, *Prevotella* is abundant in various body parts [33], and its members are related to the occurrence and development of various diseases [33, 34]. Therefore, *Prevotella sp900557255* may be an important target for predicting AA.

Since the techniques used to isolate and characterize bacterial communities are relatively developed and standardized at present, most microbiome research focuses more on the bacterial component of the human microbiome [35, 36]. The difficulties in virus isolation, nucleic acid extraction, sequencing, and pipelines analysis cause that the research on the EVs is still in its infancy [37]. In this study, it was found that *Lily virus*,* Drulisvirus*, *Felixounavirus* were highly enriched in AA, and *Felixounavirus* was an important variable in AA among these. *Felixounavirus* has strict lytic activity, and it has been shown that the phages of the *Felixounavirus* genus infect several different subtypes of *Salmonella* serosubtypes, which demonstrated their promising potential for biological control of *Salmonella* [38]. *Felixounavirus* can be a potential tool for the prediction of AA.

The effective classification and identification of microorganisms is important for many applications in microbiology [39]. It is well known that the profile and structure of the gut microbe varies among the inhabitants of a given region with ethnic differences [40]. Even if the same healthy people have different susceptibility to disease, classification discussion based on disease status alone is not accurate. Clustering is the most common application of unsupervised learning [41]. Unsupervised cluster analysis, a form of machine learning, is a common approach to identifying the subtypes of disease, and unsupervised analysis can type data according to natural patterns within and between variables [42]. Unsupervised clustering in most studies is mainly based on gut bacteria, with a handful based on enterovirus classification. In this study, we collected both gut bacteria and EVs, and then divided them into 4 subtypes by unsupervised clustering. To our satisfaction, it was found that the accuracy of the model after typing was exactly higher than that before typing, which meant that our subtypes were meaningful.

The risk prediction model plays an important role in the prediction, diagnosis, and classification of disease severity. Amin Adibi developed and validated ACCEPT model and the results proved that ACCEPT is better than using an individual’s history of acute exacerbations to predict future risk of exacerbations, especially for the accuracy of severity [43]. We previously had constructed prediction models of low-differentiated CRC based on gut bacteria and prediction models of CRC lymph node metastasis based on gut bacteria in the study of CRC, and finally determined that gut microbes can be used as a biomarker to predict low-differentiated CRC, and can also provide a new evaluation method for CRC lymph node metastasis [29, 44]. Risk prediction models were established based on characteristic bacteria and EVs in this study, which showed that CatBoost model has a good modeling effect (bacteria: discovery set: 84.62%; test set: 87.27%; virus: discovery set: 88.74%; test set: 83.33%). CatBoost models were used again to establish risk prediction models based on characteristic bacteria in 4 subtypes. The results showed that the model after typing had higher accuracy (100%, 96.34%, 100%, 98.46%, respectively) and better performance.

However, this study still has certain limitations. Firstly, based on bioinformatics analysis methods, this study revealed the differences in gut microbe between NC and AA, and established an early warning model for AA. Due to the complex interactions between individual gut microbe and their hosts, these analysis methods cannot determine whether there is a concomitant or causal relationship between gut microbe and AA. Therefore, in-depth mechanism research is expected to elucidate the relationship more accurately between them based on these data. More efforts will be devoted to explore the mechanisms of the discussion of gut microbes and AA cell mechanism by animal experiment. Secondly, the insufficient sample size of this study also limits the applicability of the research results. In the future, multicenter research is required to further verify whether this microbe can serve as promoting factors for the development of AA and to further discover the connection between these microbe and AA development. Long-term replication experiments with larger sample sizes and coverage will be conducted to validate the applicability of the 4 gut subtypes identified in this study. Finally, as an important environmental factor for AA occurrence, the gut microbe is related to various factors such as diet and economic status. However, due to the difficulties in developing evaluation indicators and collecting clinical data, as well as the limitations of research duration, these factors were not included in the model construction of this study. Therefore, more samples in future research will be included to further explore the relationship between gut microbe, EVs, and AA through metabolomics analysis methods, thus further improving the predictive performance of the model.

## Conclusion

The composition of gut microbe varies depending on disease status. In this study, the characteristics of gut microbes in AA were analyzed. The characteristic gut microbe of AA was identified. *Prevotella sp900557255*, *Megamonas funiformis*, and *Alistipes putredinis* are the common bacteria in the intestines of AA, while Drulisvirus, Lilyvirus, and Felixounavirus are the common EVs. Gut microbes were divided into 4 subtypes by unsupervised clustering. The characteristics of gut microbes in different subtypes were analyzed, and risk prediction models were established for AA-based characteristic bacteria in each subtype. The prediction results were more accurate after typing. Our study types AA and constructs risk prediction models for AA, and provides new targets and a new method for predicting AA.

### Electronic supplementary material

Below is the link to the electronic supplementary material.

Supplementary Material 1

Supplementary Material 2

Supplementary Material 3

Supplementary Material 4

## Data Availability

The datasets generated for this study can be accessed from the NCBI Sequence Read Archive (SRA) database under the accession number PRJNA1108335 (http://www.ncbi.nlm.nih.gov/bioproject/1108335) and PRJNA1108039 (http://www.ncbi.nlm.nih.gov/bioproject/1108039). The data has been released to the public.

## Declarations

### Data summary

The datasets generated for this study can be accessed from the NCBI database (https://db.cngb.org/search/project/CNP0004360/). The authors confirm that all supporting data, code, and protocols have been provided within the article or through supplementary data files.

### Impact statement

There is growing evidence that AAs are at increased risk of advanced tumors. The occurrence and development of advanced adenomas are related to many factors, among which environmental factors (including the role of gut microbes) are the most prominent. In this study, we analyzed the gut microbial characteristic of AA, established a new typing method based on bacteria and viruses, and obtained 4 types of AA. Finally, the accuracy of AA prediction was improved by typing. It provides a novel idea for establishing predictive models using characteristic microbes.
