# Supplementary figures and images for: Gut microbes on the risk of advanced adenomas
Source: BMC Microbiol. 2024 Jul 18;24:264. doi: 10.1186/s12866-024-03416-z (PMC11256391; doi:10.1186/s12866-024-03416-z)

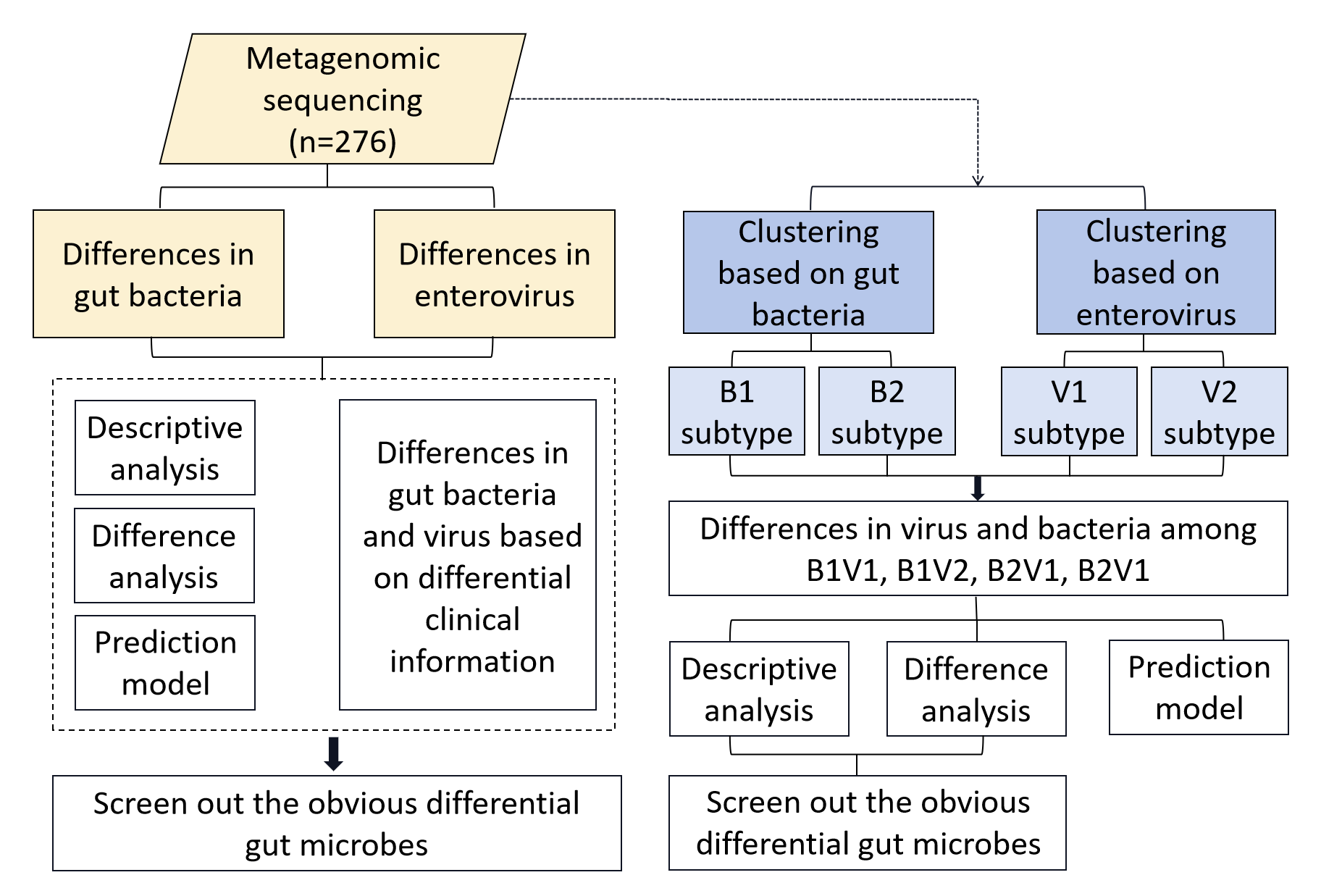

Supplement: Supplementary file 1 — Supplementary Material 1 [file 12866_2024_3416_MOESM1_ESM.png]

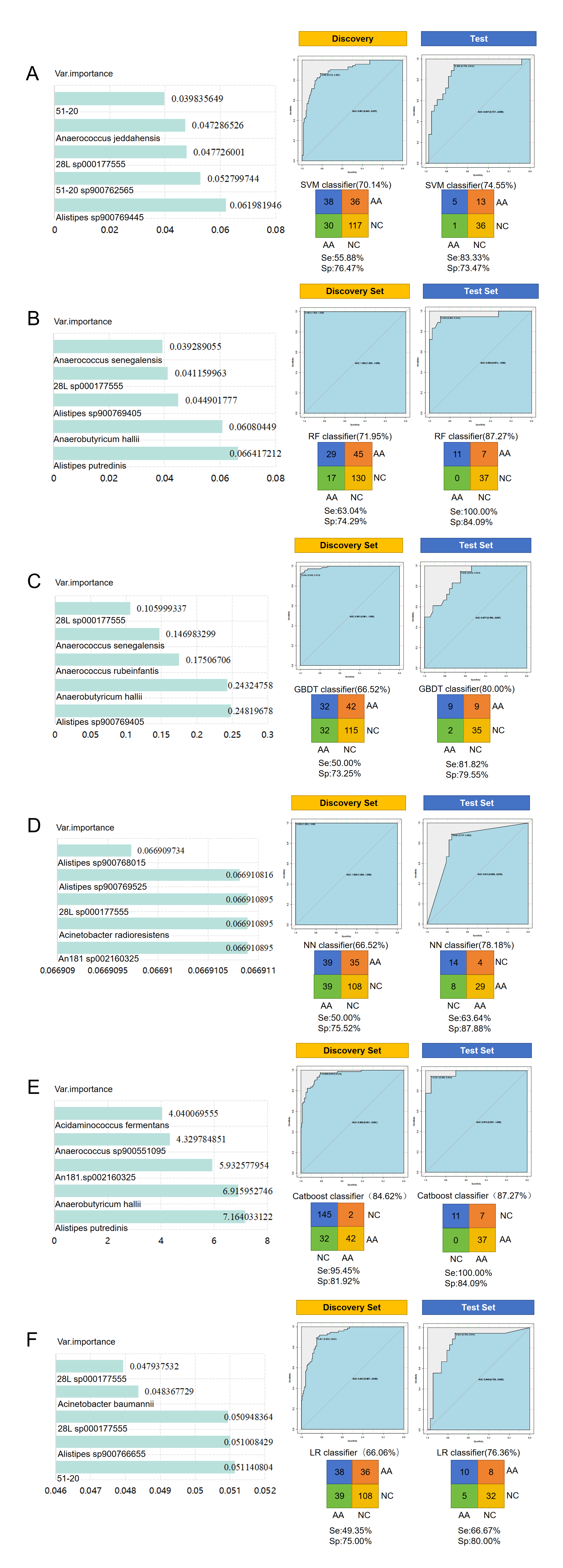

Supplement: Supplementary file 2 — Supplementary Material 2 [file 12866_2024_3416_MOESM2_ESM.png]

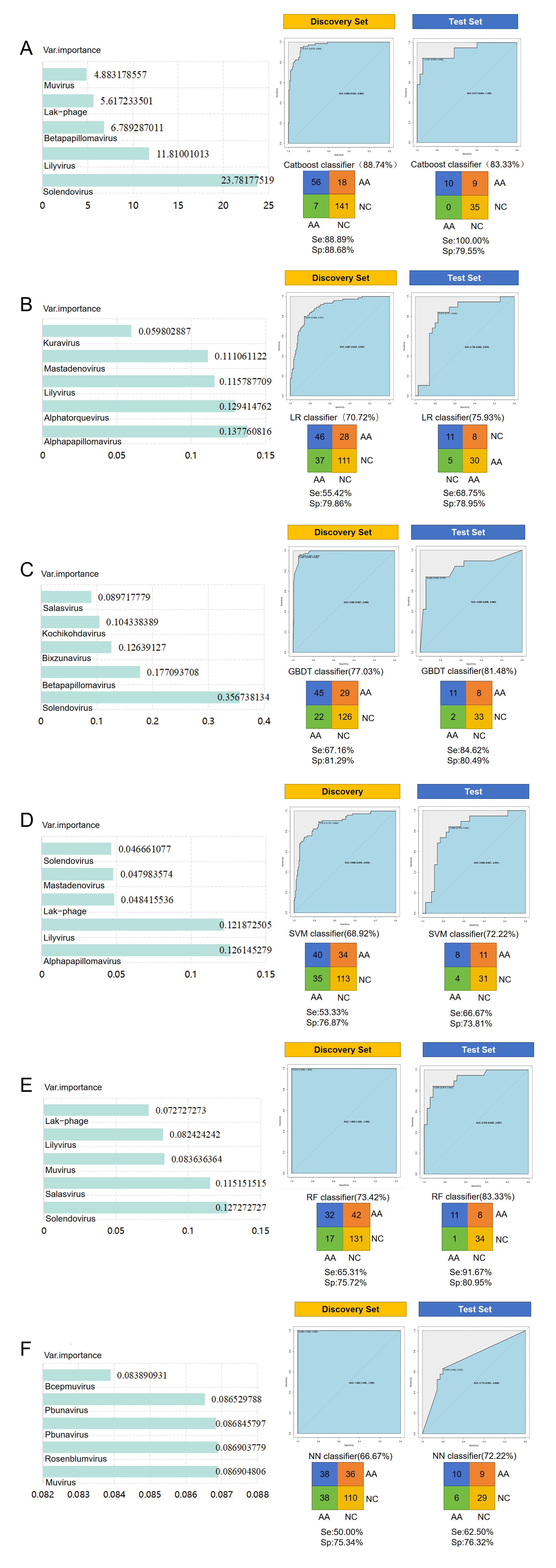

Supplement: Supplementary file 3 — Supplementary Material 3 [file 12866_2024_3416_MOESM3_ESM.png]
