# Supplementary material for: Gut microbes on the risk of advanced adenomas
Source: BMC Microbiol. 2024 Jul 18;24:264. doi: 10.1186/s12866-024-03416-z (PMC11256391; doi:10.1186/s12866-024-03416-z)
Supplement: Supplementary file 4 — Supplementary Material 4 [file 12866_2024_3416_MOESM4_ESM.docx]

**Figure S1 The flowchart of the study**

**Figure S2** **Modeling based on differential gut bacteria:** Models based on differential gut bacteria were built: SVM (A), RF (B), GBDT (C), NN (D), CatBoost (E), and LR (F).

**Figure S3** **Modeling based on differential EVs:** Models based on differential gut bacteria were built: CatBoost (A), LR (B), GBDT (C), SVM (D), RF (E), and NN (F).
